# Minimally Invasive Surgery for Giant Epidermoid Cysts of the Head and Neck Region: A Case Series

**DOI:** 10.7759/cureus.86513

**Published:** 2025-06-21

**Authors:** Torahiko Nakashima, Ryutaro Uchi, Hideoki Uryu

**Affiliations:** 1 Otolaryngology - Head and Neck Surgery, National Hospital Organization (NHO) Kyushu Medical Center, Fukuoka, JPN; 2 Otorhinolaryngology, Yamaguchi Red Cross Hospital, Yamaguchi, JPN

**Keywords:** epidermoid cyst, head and neck, infratemporal fossa, minimally invasive surgery, oral floor

## Abstract

Two distinct cases of giant epidermoid cysts in rare head and neck anatomical locations - one in the sublingual space and the other in the infratemporal fossa - are reported, highlighting the variability in presentation, imaging characteristics, and surgical management. Transoral excision of the cyst was selected for case 1 to minimize cosmetic concerns. For case 2, given the benign nature of the lesion and the patient's preference for a less invasive procedure, endoscopic marsupialization via a transnasal-transmaxillary approach was chosen. This method relieved symptoms, minimized surgical risks, and preserved quality of life, with no recurrence noted after three years. These cases underscore the importance of individualized treatment strategies for giant epidermoid cysts. While complete surgical excision remains the gold standard, minimally invasive options such as marsupialization may be appropriate in selected patients, especially when considering factors such as age, comorbidities, symptoms, and cosmetic outcomes.

## Introduction

Epidermoid cysts are benign, slow-growing lesions that arise from the proliferation of epidermal cells within a cystic cavity filled with keratinous material. Although they can occur anywhere on the body, they are commonly found in the head and neck region, including the scalp, face, neck, postauricular area, and submandibular region [1-3]. The incidence of epidermoid cysts in the head and neck region has been reported to be approximately 7%, with a lower incidence of about 1.6% specifically in the oral cavity [3]. These lesions are typically diagnosed as small, subcutaneous nodules, and surgical excision is usually straightforward. However, in rare instances, giant epidermoid cysts - such as lesions exceeding 5 cm in diameter - can develop in atypical or deep anatomical locations within the head and neck. Surgical treatment is widely recognized as the standard approach. However, certain cases may present unique challenges due to factors such as tumor size, proximity to critical anatomical structures, and the potential for significant impact on the patient's quality of life based on their location.

The floor of the mouth is one of the regions where an epidermoid cyst can grow significantly, as these cysts typically present as painless swellings in the submental region and often do not affect speech or swallowing until they reach a large size [4]. Two main surgical approaches have been reported: open excision via the neck [2,5,6] and the intraoral approach [4,7,8]. From a cosmetic standpoint, the intraoral route is preferred. However, this technique offers limited surgical exposure, increasing the risk of injury to adjacent structures and potentially making complete resection more difficult. In contrast, open surgery is sometimes recommended for complicated cases, particularly when the cyst's location poses challenges for intraoral access [2].

In contrast, epidermoid cysts in the infratemporal fossa are extremely rare. There are only a few reported cases, including a case that was managed by an approach combined with zygoma resection [9]. Currently, the optimal surgical strategy for treating infratemporal epidermoid cysts has yet to be clearly established.

In this review, we present two cases of giant epidermoid cysts located in the floor of the mouth and the infratemporal fossa. We discuss surgical management strategies, with particular emphasis on minimally invasive approaches designed to optimize both functional and aesthetic outcomes while minimizing morbidity.

## Case presentation

Case 1

**Figure 1:**
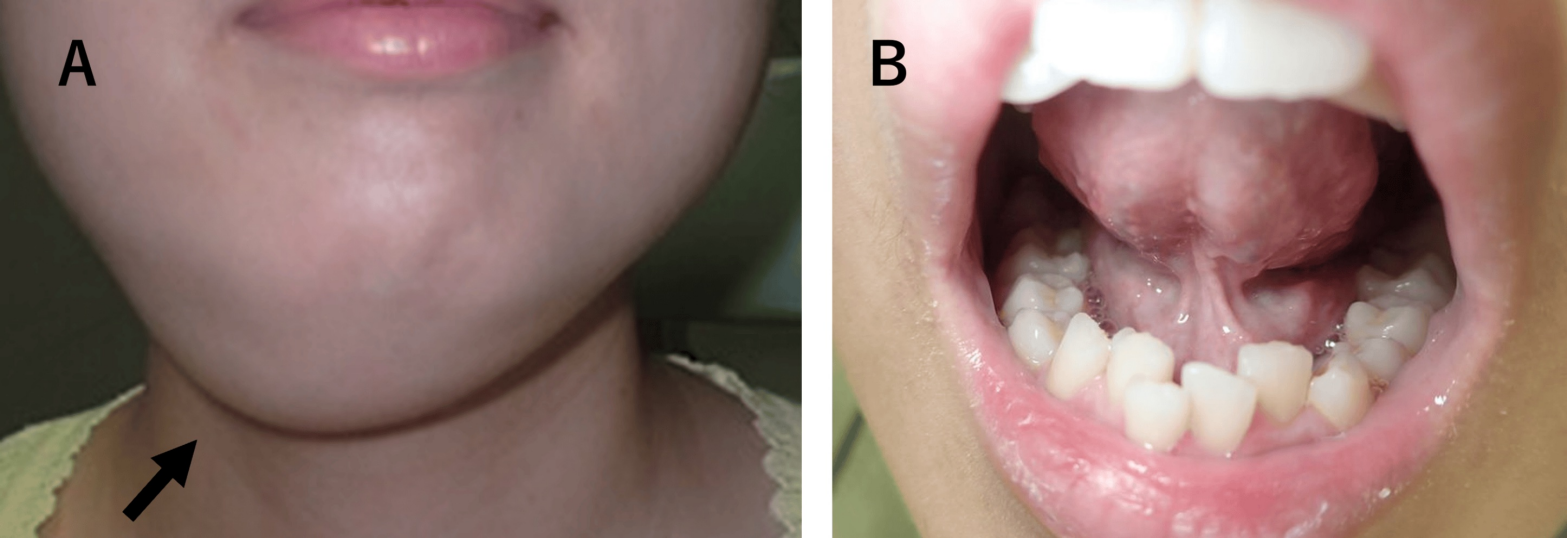
Clinical images of Case 1. (A) Extraoral view showing swelling (arrow) in the submental region. (B) Intraoral examination reveals no evident swelling of the floor of the mouth.

A 30-year-old female presented with a slowly enlarging, painless swelling in the submental region. The mass had been progressively increasing in size for approximately five years prior to presentation. The patient denied any neurological symptoms, such as tongue palsy, and any difficulties with swallowing. She had attributed the swelling to fatty tissue, which caused her delay in seeking medical attention. On physical examination, a soft, fluctuant, and painless swelling was observed in the submental region (Figure 1A). There was no palpable cervical lymphadenopathy, and the floor of the mouth appeared normal, with no evidence of swelling (Figure 1B).

Imaging

**Figure 2:**
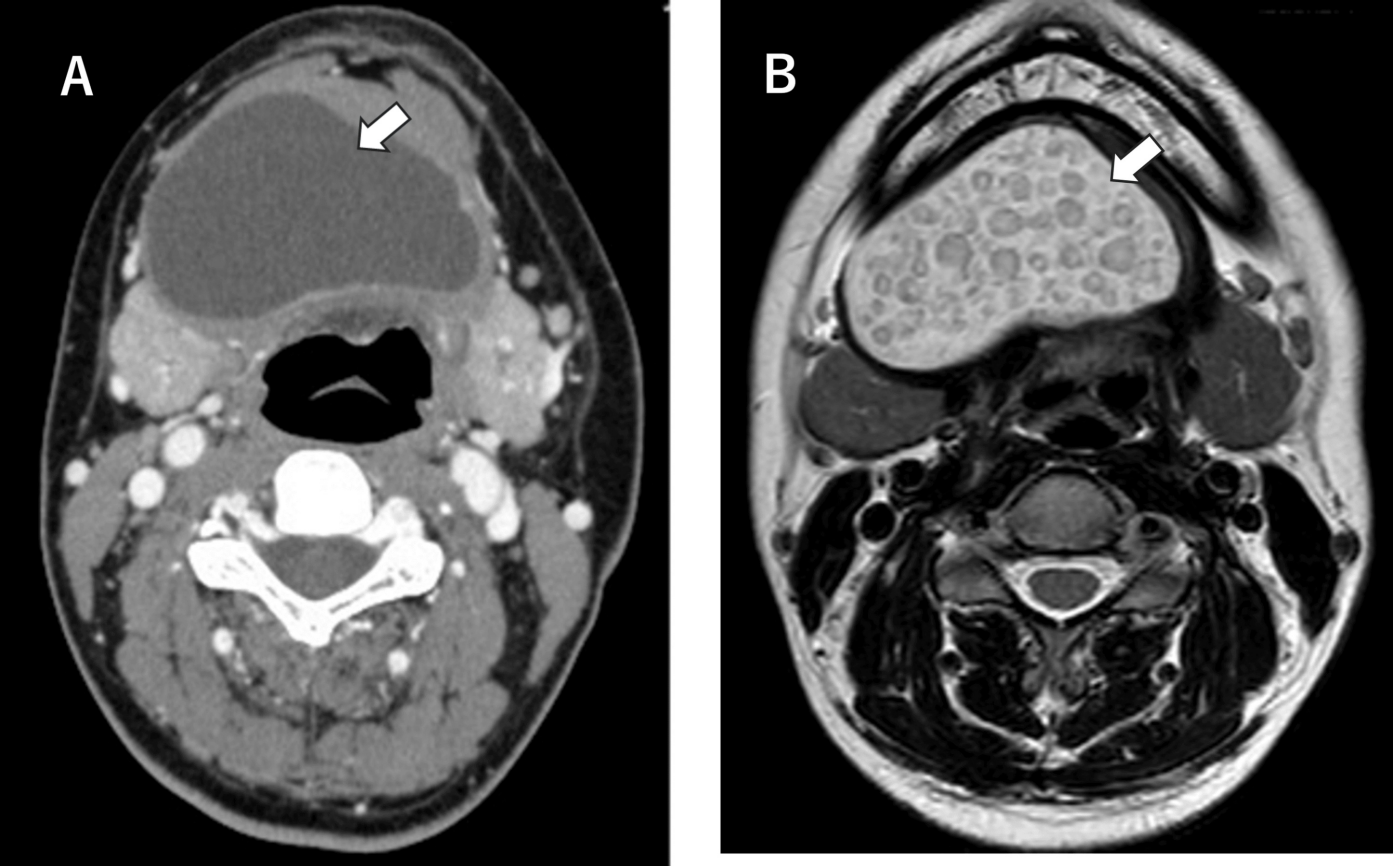
Preoperative computed tomography (CT) (A) and T2-weighted MRI axial view (B) of Case 1 demonstrating the extent of the cyst (arrows). The MRI shows the characteristic "sac of marbles" sign, a radiologic hallmark suggestive of an epidermoid cyst.

Differential diagnosis included epidermoid cyst, ranula, mucocele, and minor salivary gland tumors. Contrast-enhanced CT revealed a thick-walled cystic lesion measuring 62 × 36 × 43 mm located in the sublingual space (Figure 2A). Magnetic resonance imaging (MRI) revealed a well-circumscribed, non-enhancing cystic lesion showing low signal intensity on T1-weighted images and high signal intensity on T2-weighted images, without restricted diffusion. Notably, the lesion demonstrated the characteristic “sac of marbles” sign [10], a radiologic hallmark suggestive of an epidermoid cyst (Figure 2B).

Surgical Management

**Figure 3:**
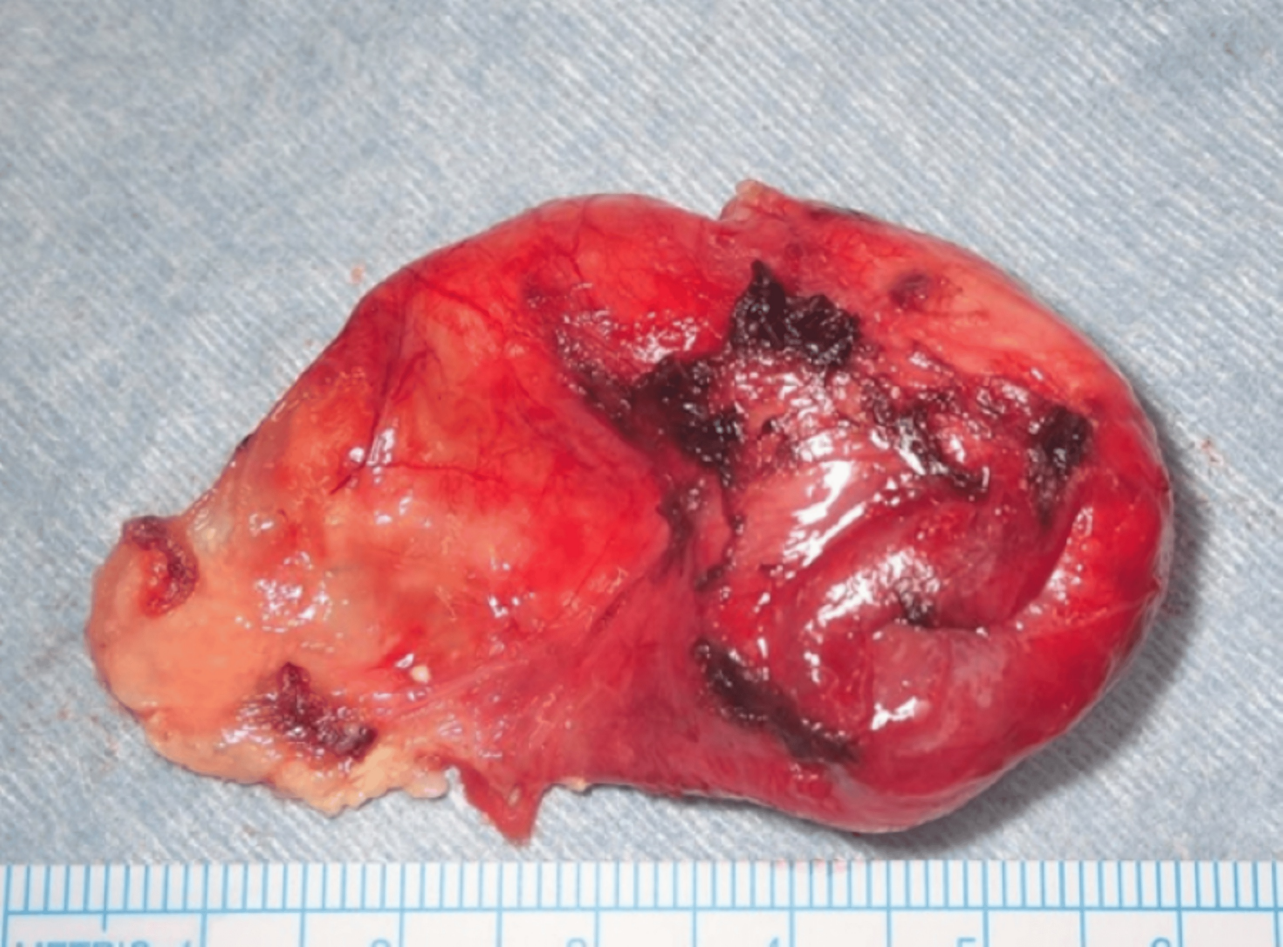
Macroscopic view of the resected cyst of Case 1.

Given the patient’s cosmetic concerns, a transoral approach was selected. Under general anesthesia with nasal intubation, an incision was made on the floor of the mouth, and the capsule of the lesion was identified. Care was taken to avoid damage to surrounding structures. Due to the large size of the tumor, transoral resection was deemed challenging. Therefore, partial decompression of the tumor contents was performed, followed by complete excision along the capsule (Figure 3). There were no intraoperative or postoperative complications, and the patient had an uneventful recovery. At the three-year follow-up, there has been no evidence of recurrence.

Histopathology

**Figure 4:**
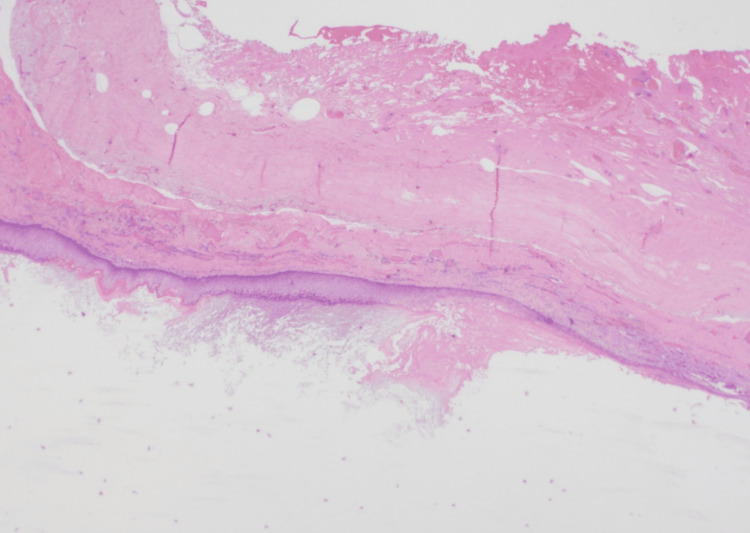
Histologic study image (Case 1) (20X magnification). HE stain showing a cyst cavity filled with keratin. The cystic wall is lined by squamous epithelium.

Postoperative histologic examination confirmed the diagnosis of an epidermoid cyst, showing a cystic cavity lined by keratinized stratified squamous epithelium without skin appendages (Figure 4).

Case 2

A 67-year-old woman was transferred to our institution for further evaluation and management of persistent left facial pain that gradually developed over the preceding six months. Initial assessment at the referring hospital had revealed a cystic lesion in the infratemporal fossa. On physical examination, the patient had no visible facial or head swelling, no trismus, with no additional neurological deficits observed. Differential diagnosis included dermoid/epidermoid cyst, abscess, benign and malignant neoplasms.

Imaging

**Figure 5:**
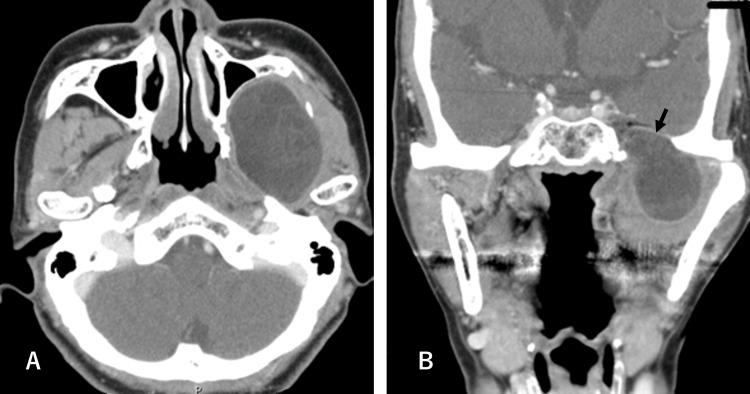
Preoperative computed tomography (CT) scans of Case 2. (A) Axial and (B) coronal views reveal a well-defined cystic lesion in the left infratemporal fossa. Thinning of the skull base bone is observed (arrow in B), likely due to the expansion of the cyst wall.

**Figure 6:**
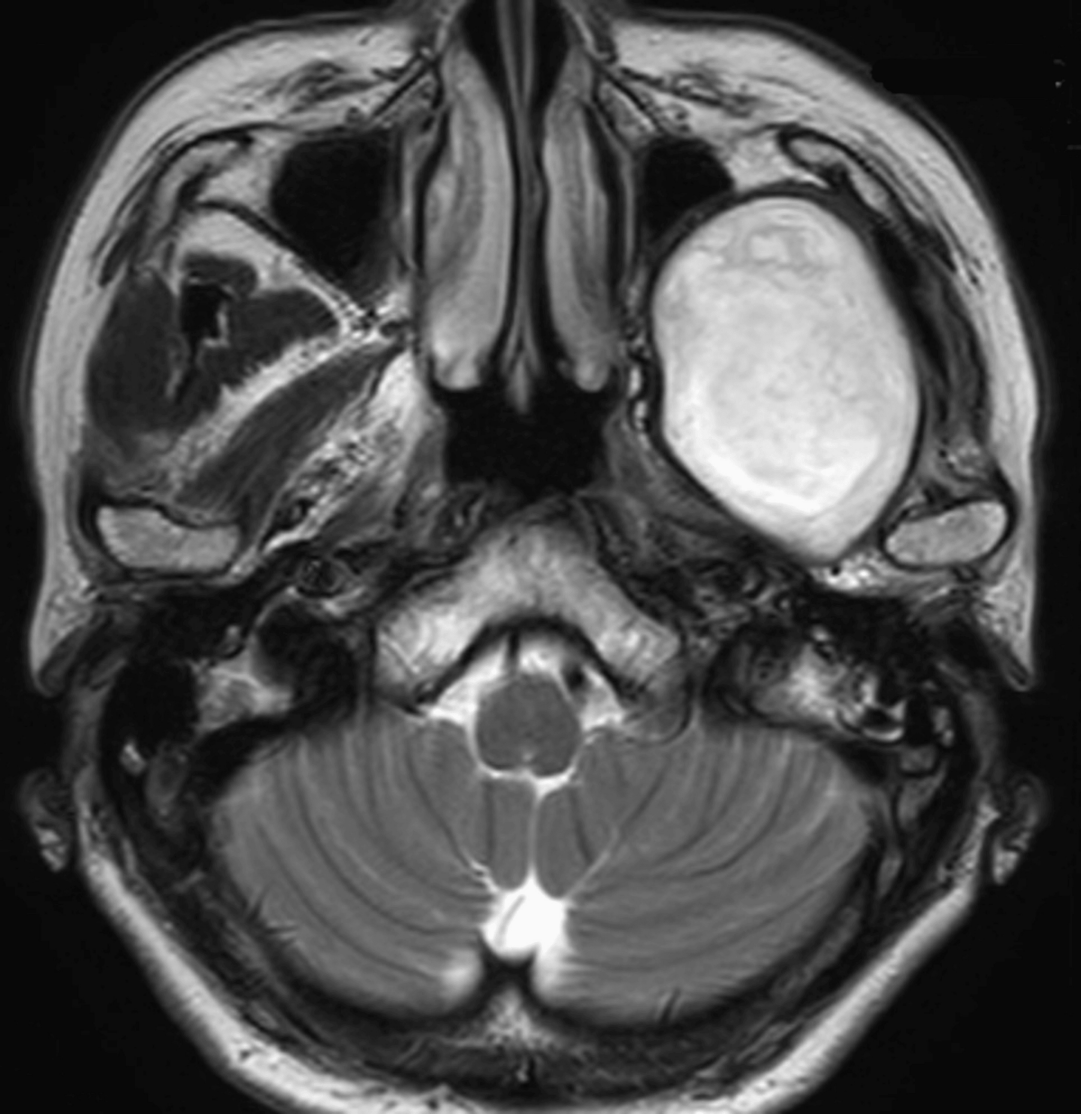
T2-weighed MRI in the axial view of Case 2.

Contrast-enhanced CT (Figures 5A-5B) and MRI (Figure 6) revealed a 54 × 43 × 50 mm well-defined lesion, with no significant post-contrast enhancement, cystic mass located in the left infratemporal fossa. The bone at the skull base was thinned due to the expansion of the cyst wall, but there was no evidence of infiltration into the brain (Figure 5B).　　

Diagnostic imaging raised suspicion for an epidermoid cyst, and a puncture of the lesion was subsequently performed. The aspirated contents were keratinous in nature, consistent with the diagnosis of an epidermoid cyst.

Surgical Management

**Figure 7:**
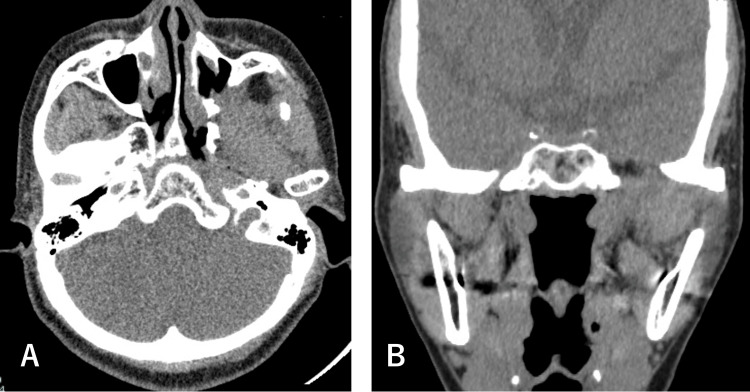
Postoperative CT scans of Case 2 at three-year follow-up. (A) Axial view and (B) coronal view demonstrate the effective management of the swelling, with no evidence of recurrence.

Two surgical options were proposed: complete excision via a temporal approach or marsupialization via an endoscopic transnasal-transmaxillary approach. Given the high likelihood of the lesion being benign and with consideration for postoperative quality of life, the patient elected to proceed with the less invasive option. An endoscopic maxillary antrostomy was performed, followed by partial resection of the cystic lesion and marsupialization. The postoperative course was uneventful, with marked improvement in symptoms and no complications. At the three-year follow-up, there has been no evidence of recurrence (Figure 7).

## Discussion

Epidermoid cysts result from the inclusion of ectodermal elements during embryonic development. The etiopathogenesis of epidermoid cysts is thought to involve either congenital inclusion of ectodermal elements during embryogenesis or post-traumatic/surgical implantation of epidermal cells into deeper tissues. In the head and neck region, congenital epidermoid cysts are believed to arise from ectodermal remnants trapped during midline fusion of the branchial arches, particularly in areas such as the floor of the mouth or sublingual space. Epidermoid cysts most commonly present in young adults, typically between the second and fourth decades of life. The cysts are filled with debris containing keratin from the desquamation of their epithelial lining [11].

In the floor of the mouth, which is a relatively common region of epidermoid cysts within the head and neck [12,13], they may cause visible submental swelling and can be mistaken for other midline neck masses, including dermoid cysts or ranulas. MRI, particularly T2-weighted imaging, is valuable for preoperative assessment, with the “sac of marbles” sign being pathognomonic [10].

Surgical excision remains the definitive treatment; however, it may adversely affect patients' quality of life, and its indication should therefore be carefully evaluated.

In Case 1, a transoral approach was chosen, offering the advantage of avoiding external wounds and scarring - an important consideration, particularly in young patients. Compared to the cervical approach, it has clear cosmetic benefits. However, the transoral approach provides limited exposure, and care must be taken to excise the cyst along its capsule to avoid injury to adjacent structures such as the lingual nerve and Wharton’s duct.

Radiological examination using MRI and CT is essential for evaluating the size and extent of the cyst. When selecting a surgical approach, several factors should be considered, including the patient’s age, gender, cyst size, location, and personal preferences. Sejati et al. recommended a cervical approach when the cyst is located below the mylohyoid muscle, as this offers better access and visualization for complete excision [4].

In Case 2, a transnasal-transmaxillary approach was selected for the fenestration procedure instead of the subtemporal route. The cyst was fenestrated into the maxillary sinus, resulting in complete resolution of the patient’s swelling. Notably, there have been no signs of recurrence over the past three years. Sendjaja et al. reported a surgical approach for infratemporal epidermoid cysts involving a frontotemporal craniotomy combined with zygomatic resection [9]. In our case, surgical intervention prioritized symptom relief and preservation of quality of life over complete cyst excision. Although our treatment strategy did not aim for total resection, creating a drainage route from the cyst to the nasal cavity proved sufficient to alleviate symptoms and sustain the patient’s quality of life. This minimally invasive approach reduced surgical risks and avoided potential complications associated with more extensive procedures, making it a viable option in selected cases.

Although extremely rare, malignant transformation of epidermoid cysts has been reported. Most of the reported malignant cases are from intracranial epidermoid cysts [14]. Histopathological confirmation is essential to rule out rare malignant transformation. Given the potential for serious complications, it is the responsibility of surgeons to provide patients with accurate and comprehensive information regarding the nature of the disease. This includes discussing the prognosis, treatment options, and possible outcomes. Treatment strategies should be individualized, taking into careful consideration the patient’s symptoms, general health status, age, and personal preferences. Shared decision-making plays a critical role in ensuring that the selected treatment aligns with the patient’s values and clinical needs.

## Conclusions

Epidermoid cysts are rare, benign congenital lesions arising from ectodermal inclusions. They account for approximately 7% of head and neck cysts, with only 1.6% occurring in the oral cavity and even fewer reported in the infratemporal fossa. When located in atypical sites such as the floor of the mouth or the infratemporal fossa, giant cysts can present with a diverse range of symptoms. In this report, we present two cases of giant epidermoid cysts involving these uncommon locations. Considering the impact on patients’ quality of life, minimally invasive surgical approaches were selected, resulting in successful outcomes.

The definitive treatment for epidermoid cysts is surgical excision. However, minimally invasive techniques, including marsupialization, may be appropriate in select cases where surgical risk, comorbidities, or quality-of-life considerations play a significant role. Although malignant transformation is rare, appropriate counseling and long-term follow-up are essential. Treatment should be individualized through a shared decision-making process.
